# Study on Analysis and Sedimentation of Alumina Nanoparticles

**DOI:** 10.3390/ijerph16030510

**Published:** 2019-02-12

**Authors:** Xuehong Zheng, Yuehan Li, Ding Chen, Airong Zheng, Qikang Que

**Affiliations:** 1 College of Ocean and Earth Sciences, Xiamen University, Xiamen 361102, China; bubbleno.1@163.com (Y.L.); chending@xmu.edu.cn (D.C.); arzheng@xmu.edu.cn (A.Z.); 2 Key Laboratory of Marine Chemistry and Application (Xiamen University), Fujian Province University, Xiamen 361102, China; 3 SGS-CSTC Standard Technical Services Co., Ltd., Xiamen Branch, Xiamen 361101, China; ben.que@sgs.com

**Keywords:** alumina nanoparticles, analysis method, sedimentation, pH, salinity

## Abstract

Dispersion and aggregation behavior of nanoparticles in aquatic environment may be affected by pH, salinity, and dissolved organic matter, which would change its ecological risk. Effects of time, power and temperature on the alumina nanoparticles (nano-Al_2_O_3_) ultrasonic dispersion in water were discussed. Al_2_O_3_ had a best ultrasonic dispersion for 30 min at 105 W and 30 °C. The concentration of Al_2_O_3_ could be measured by ultraviolet (UV) spectrophotometer, and the method was efficient and accurate. Furthermore, the sedimentation rate of Al_2_O_3_ was related to pH, salinity, and its concentration in the artificial seawater. When pH was 7.31, approaching the isoelectric point of Al_2_O_3_, they aggregated and settled fastest. Settlement coefficient (*k*) of Al_2_O_3_ increased by 3 and 2.7 times while the salinity and its concentration increased. The sedimentation rate was higher in natural seawater than that in artificial seawater. All results indicated that nano-Al_2_O_3_ would be removed in aquatic environment.

## 1. Introduction

With the rapid development of nanotechnology, nanoparticles (NPs) are discharged into the aqueous environment by waste water, river runoff or atmospheric deposition. Because of its unique physical and chemical properties, they may combine with natural organic matter, inorganic nutrients and heavy metal ions in seawater, then remove from the water by the reunion and sedimentation, or fed by the aquatic organisms, and affect the marine ecological environment [1,2,3,4,5]. In recent years, more and more scientists focused on the ecological toxicity of nanomaterials to aquatic organisms. NPs accumulation mainly occurred in digestive tract and gills of marine invertebrates. They interacted with plasma proteins, forming a protein corona that could affect particle uptake and toxicity in target cells in a physiological environment [6]. In marine systems, metal oxide nanoparticles could absorb to micro-organisms with potential for trophic transfer following consumption [7]. The photosynthetic activity of microalgae, after addition of ZnO NPs, decreased progressively due to stress induced by the presence of the nanoparticles in the culture medium [8]. The alpha-aluminum oxide particles showed lower toxicity than the gamma-phase aluminum oxide, indicating that the crystalline structure of nanoparticles may influence their toxicological impact on biota [9]. Therefore, it is necessary to establish a nanomaterial analysis method to predict its potential threat to the environment.

Nano-Al_2_O_3_ is one of the most used ultrafine powders in the world, and the market is increasing by 5.8% [10]. It has a completely new structure that has unique properties, so that it is widely used in the magnetism, electricity, optics, catalysis and chemical sensing, and so on. While a large number of nano-Al_2_O_3_ have been manufactured and used, they impose negative influence on microorganisms, and the ecosystem confronts more potential risk [11]. There were a few researches about the existence, migration of nanomaterials (NMs), and their interaction with coexistence material in environment. NMs may undergo a wide range of weathering or ‘aging’ processes that will alter their surface chemistry and, therefore, transport and potential exposure routes. These transformations occur through processes such as redox reactions, interactions with organic macromolecules such as natural organic matter (NOM) or cellular material, dissolution, or adsorption of known pollutants (e.g., As, Hg, polychlorinated biphenyls, polycyclic aromatic hydrocarbons) [12]. For example, Fe_2_O_3_ NPs had the highest fractionation factor between Th-234 and Pa-233, while SiO_2_ NPs had the lowest, following the order of Fe_2_O_3_ > CaCO_3_ > Al_2_O_3_ > TiO_2_ > SiO_2_ [13]. By the influence of pH (6.0–9.0), natural organic matter (NOM) (0–10 mg C/L) and ionic strength (IS) (1.7–40 mmol/L), 14 nm CeO_2_ NPs could aggregate in the size between 200 and 10,000 nm. Increasing pH and IS enhanced aggregation, while increasing NOM decreased mean aggregate sizes [14]. The NPs coating by humic acid induced disaggregation behavior in the ZnO nanoparticles and decreased the aggregate size in parallel to increasing humic acid concentrations [15].

Nanoparticles have small size, large specific surface area, and thermodynamic instability in the aqueous system, coupled with the electrostatic force between particles and van der Waals force, so they may aggregate to larger particles, which affects the analysis [16]. Therefore, the accuracy of nanoparticles content analysis depends on whether they can disperse uniformly in the medium [17]. In the past years, dynamic reaction cell inductively coupled plasma-mass spectrometry was used to investigate the behavior of aqueous suspensions of SiO_2_ nanoparticles in terms of agglomeration and dissolution [18]. Furthermore, high-throughput screening methods using UV spectrophotometry were employed to characterize the concentration of silver NPs and presented their sedimentation behaviors in natural freshwaters, synthetic seawater, and simulated estuarine waters. It is a simple and rapid approach for detection and quantification [19,20].

Therefore, by ultrasonic dispersion technology and UV spectrophotometry [21], the effects of ultrasonic time, power and temperature on the dispersion of nano-Al_2_O_3_ suspension were studied. In the optimal dispersion condition, the analysis method of nano-Al_2_O_3_ was discussed, and the test of the additive recovery and precision were carried out to verify the reliability. Furthermore, simulated experiments were carried out to study effects of pH, salinity, and concentration on the sedimentation of nano-Al_2_O_3_, and the difference of its settlement in artificial seawater and in natural seawater medium was compared. All results could provide technical basis and experimental data for environmental behavior of nanoparticles.

## 2. Materials and Methods

### 2.1. Materials

80 nm Al_2_O_3_ powder (99.99%, Beijing dekedaojin technology Co., Ltd., Beijing, China) was used, which should be dried at 60 °C to constant weight before use. Artificial seawater (ASW) was prepared according to the artificial seawater formula of Bidwell and Spotte [22], salinity(*S*) = 31.50, pH = 8.00. Natural seawater (NSW) was collected from Xiaogao Fishing Port in Fujian Province, China. The concentration of total organic carbon (TOC) was 117 μmol/L, analyzed by TOC-VCPH total organic carbon analyzer (Shimadzu Co., Kyoto, Japan). *S* was 29.8 at 25 °C by Cond 3110 conductivity meter (WTW Co., Munich, Germany). pH was 7.95 at 25 °C by pH meter (WTW Co., Munich, Germany). The absorbances (*A*) of nano-Al_2_O_3_ suspensions were test by UV mini-1240 UV spectrophotometer (Shimadzu Co., Kyoto, Japan).

### 2.2. Analysis of Nano-Al_2_O_3_ in Artificial Seawater

#### 2.2.1. Study of Optimum Dispersion Conditions

Ultrasonic dispersion experiments of 100.0 mg/L nano-Al_2_O_3_ ASW suspensions were set as Table 1. Three groups with different conditions were used to observe the effect of ultrasonic time (*t*), ultrasonic power (P), and ultrasonic temperature (T) on dispersion. After the ultrasonic dispersion, the absorbance of suspension was test at 340 nm.

#### 2.2.2. Parameters of UV Spectrophotometry

The absorbances of 10 blank parallel samples were tested for method detection limit estimation [23]. The equation is

*C_DL_* = 3*δ*/*m* (1)

In Equation (1): *C_DL_* is method detection limit, *δ* is standard deviation of absorbance, *m* is the slope of the standard curve.

5–40 mg/L nano-Al_2_O_3_ were added to the 9.00 mg/L, 19.21 mg/L, 40.52 mg/L nano-Al_2_O_3_ suspensions, respectively to obtain the recovery of the experiments.

10.00 mg/L, 50.00 mg/L, and 100.0 mg/L nano-Al_2_O_3_ suspensions, were tested with six parallel samples respectively, and the relative standard deviation (RSD) was calculated by Excel.

#### 2.2.3. Method Comparing between Gravimetry and UV Spectrophotometry

Gravimetry [24] and UV spectrophotometry were compared in the concentration analysis of nano-Al_2_O_3_ suspensions. 10.00 mg/L, 50.00 mg/L, and 100.0 mg/L suspensions were filtrated by 0.45 μm polycarbonate membranes with given dry weight. The membranes were dried in 45 °C for 8 h before storing in a dryer to constant weight. According the variation of membranes weight and volumes of suspension, concentrations could be calculated. At the same time, all the suspensions were tested by UV spectrophotometry.

### 2.3. Nano-Al_2_O_3_ Settlement Experiments

The experiments were set as Table 2 to study the effects of pH, *S*, and concentration on nano-Al_2_O_3_ sedimentation in ASW. pH of ASW was controlled by adding hydrochloric acid or sodium hydroxide. After the ultrasonic dispersion, the absorbances of nano-Al_2_O_3_ suspensions were tested every 5 min. Changing ratios of absorbance with time (*A_t_*/*A*_0_) were compared, while *A_t_* was absorbance at time *t*, *A*_0_ was absorbance at *t* = 0.

In addition, the settlements of 100.0 mg/L nano-Al_2_O_3_ suspensions in ASW and NSW were investigated. pH and salinity of ASW were the same as that of NSW.

All the sedimentation can be fitted with a first-order dynamic model. By the software SigmaPlot, the fitting equation is

(2) $$lnA_{t}=-kt+lnA_{0}$$

In the equation: *A*_0_ and *A_t_* respectively represent the absorbance of suspensions at time 0 and *t*, unit: mg/L; *k* represents the settlement coefficient, unit: min^−1^; *t* means time, unit: min.

## 3. Results and Discussion

### 3.1. Analysis Results of Nano-Al_2_O_3_ in Artificial Seawater

#### 3.1.1. Optimal Dispersion Conditions

**Figure 1:**
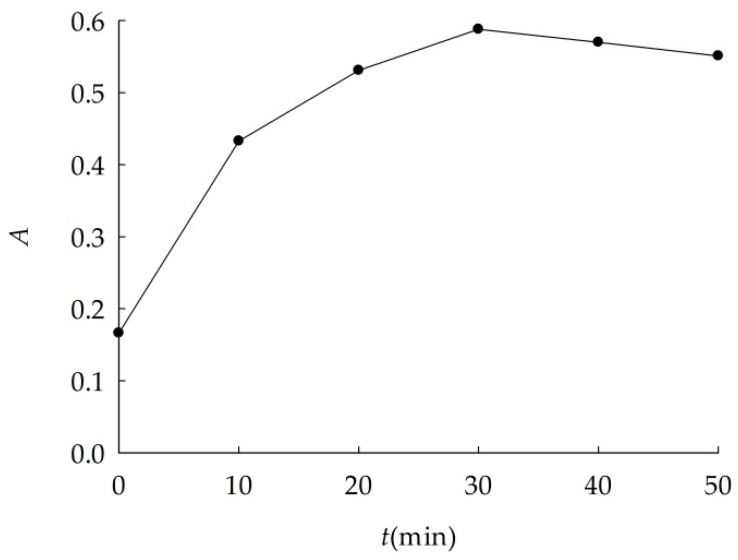
Absorbance (*A*) curve of nano-Al_2_O_3_ suspension at different ultrasonic time.

Results of experiments in Table 1 indicated optimal dispersions conditions. As shown in Figure 1, absorbance of suspension increased with ultrasonic time at first, then decreased slightly. Absorbance maximum could be found when the ultrasonic time was 30 min. Thus, the optimal ultrasonic time was 30 min.

**Figure 2:**
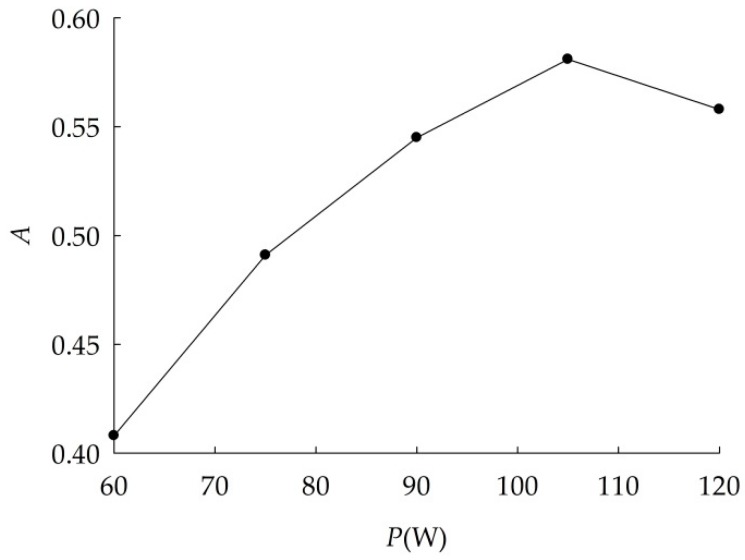
Absorbance (*A*) curve of nano-Al_2_O_3_ suspension at different ultrasonic power.

Effects of ultrasonic power on dispersion were shown in Figure 2. Absorbance of suspensions increased with ultrasonic power linearly from 60 W to 105 W, then decreased. When the ultrasonic power was 105 W, absorbance was the maximum, indicating that nano-Al_2_O_3_ could disperse very well. Therefore, the optimal ultrasonic power was 105 W.

**Figure 3:**
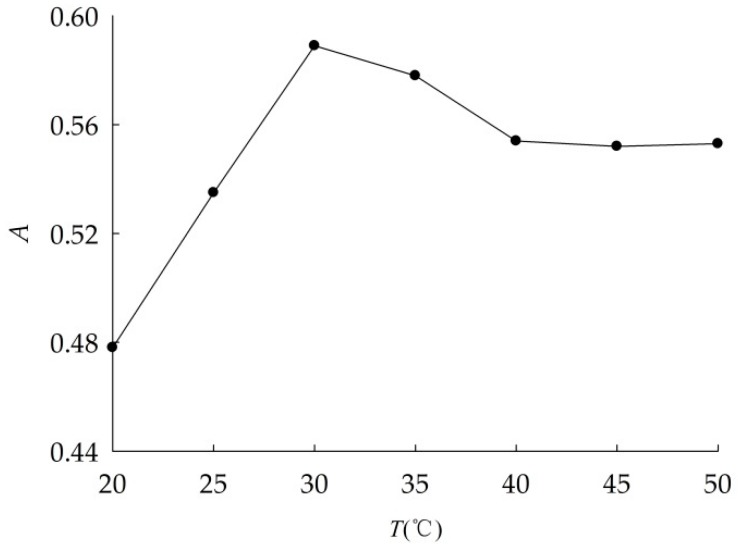
Absorbance (*A*) curve of nano-Al_2_O_3_ suspension at different ultrasonic temperature.

The absorbance of nano-Al_2_O_3_ suspensions at different ultrasonic temperatures were shown in Figure 3. When ultrasonic temperature was 30 °C, nano-Al_2_O_3_ dispersed the best, thus it is the optimum ultrasonic temperature.

#### 3.1.2. Parameters of UV Spectrophotometry

Method detection limit was set to be 0.93 μg/L based on blank samples which were tested at 340 nm after ultrasonic dispersing at above optimum conditions, showing that this method was suitable for detecting trace nano-Al_2_O_3_ in water. Recovery of nano-Al_2_O_3_ by UV spectrophotometry was shown in Table 3, it was 97.4–106.4%. While the initial concentration or additive increase, recovery was stable, indicating that UV spectrophotometry was applicable for nano-Al_2_O_3_ analysis.

RSD of 10.00, 50.00, and 100.0 mg/L nano-Al_2_O_3_ suspensions were 3.1, 5.5, and 2.2%, respectively (Table 4), the relative deviations were 13.7, 0.7, and 2.3%, respectively. All results showed that UV spectrophotometry was reliable.

#### 3.1.3. Difference between Gravimetry and UV Spectrophotometry

Method comparing results were shown in Table 5. The relative deviation of gravimetry and UV spectrophotometry were 9.3–26.0% and 0.5–13.2% respectively, which means that the latter was better. In addition, the analysis time of UV spectrophotometry was less than one hour, and that of gravimetry was about one day. Therefore, UV spectrophotometry was superior.

In a word, ultrasonic dispersing nano-Al_2_O_3_ suspension for 30 min at 30 °C and 105 W, then testing at 340 nm, was an effective and reliable.

### 3.2. Results of Settlement Experiment of Nano-Al_2_O_3_

#### 3.2.1. Effects of pH

**Figure 4:**
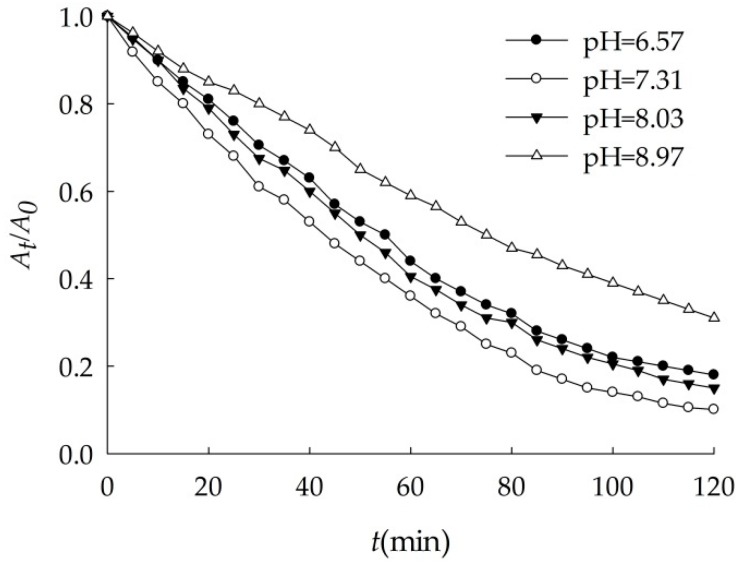
Effects of pH on the sedimentation of nano-Al_2_O_3_ (*S* = 31.5).

Sedimentation of nano-Al_2_O_3_ as a function of pH was shown as Figure 4. 100.0 mg/L nano-Al_2_O_3_ aggregated and settled in four different pH suspensions to some degree.

As shown in Table 6, the highest of settlement coefficient (*k*) was 0.021 min^−1^ when the artificial seawater pH = 7.31. The smallest was 0.010 min^−1^ when pH = 8.97, so the suspension was the most stable. The intermediate was 0.016 and 0.019 min^−1^ when pH was 6.57 and 8.03. The rules were found in other papers. The influence of pH on the sedimentation rates of alumina followed the order of 7.3 > 5 > 2 [25]. The stability of alumina suspensions decreases on approaching the isoelectric point from either side of pH [26].

The surface potential of nanoparticles is one of their important properties, and it has important influence on the dispersion of nanoparticles in solution [27]. The greater the absolute value of surface potential, the larger the electric double layer thickness of the nanoparticles, which lead to the electrostatic repulsion greater between particles, and suspension was more stable [28,29,30]. The isoelectric point of nano-Al_2_O_3_ was around 7.10 [31,32]. When pH < 7.10, nano-Al_2_O_3_ surface was positively charged. Because of the effects of static charges attracting with each other, electric double layer formed. The lower pH, the greater the thickness of electric double layer. When pH > 7.10, the surface of the particle was negatively charged, it easily adsorbed positive ions, which also formed a double layer. As pH increased, the double layer thickness increased. When the pH value approached to the isoelectric point of nano-Al_2_O_3_ particle, Al-OH appeared and particles were electrically neutral. The main force of the van der Waals attraction between particles caused them to aggregate and settle because of the chaotic movement [33]. The settlement behavior of nano-Fe_3_O_4_ in the environment also revealed the same pattern [34].

Therefore, the suspension pH = 7.31, which is near the electric point of nano-Al_2_O_3_, the double layer thickness was small. The particle repulsion potential energy was weak, so the nano-Al_2_O_3_ was prone to aggregate in the water. The suspension pH = 8.97, the double layer thickness of nano-Al_2_O_3_ was the largest, and the particles were difficult to aggregate. When the pH of the suspension was 6.57 and 8.03, the absolute value of the surface potential was similar so that they had the same sedimentation rate.

#### 3.2.2. Effects of Salinity

**Figure 5:**
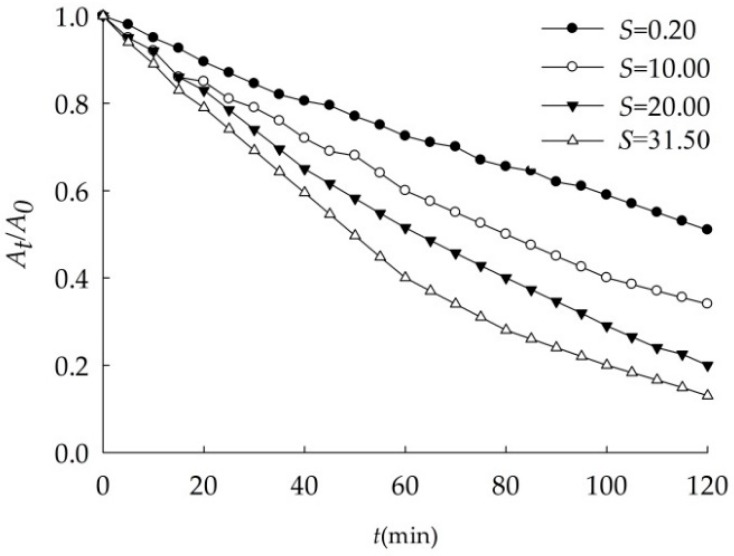
Effects of salinity on the sedimentation of nano-Al_2_O_3_ (pH = 8.00).

As shown in Figure 5 and Table 7, the sedimentation of 100.0 mg/L nano-Al_2_O_3_ changed with salinity. The higher the salinity the faster it settled. When salinity increased from 0.20 to 31.50, *k* increased to three times, showing the settling velocity of nano-Al_2_O_3_ was positively related with salinity.

In general, with the increase of salinity, solution ionic strength increases, the potential on the surface of the nanoparticles also increases [29,35], and electrolyte in the water affects the electric double layer thickness of the nanoparticles. For the same electrolyte, the higher the concentration, the greater the compression of the electric double layer of nano-Al_2_O_3_, the smaller the electrostatic repulsion between particles, and eventually lead to particle size increasing and settling [27,36].

#### 3.2.3. Effects of Nano-Al_2_O_3_ Concentration

**Figure 6:**
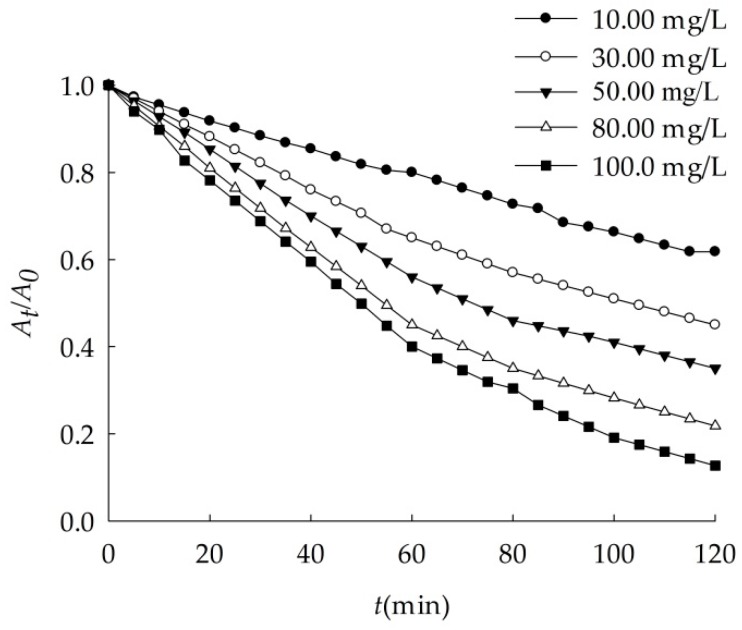
Effects of concentration on the sedimentation of nano-Al_2_O_3_ (*S* = 31.5, pH = 8.00).

When nano-Al_2_O_3_ concentration changed, its sedimentation rate varied as Figure 6. The higher nano-Al_2_O_3_ concentration, the larger the sedimentation rate. As shown in Table 8, when the concentration increased from 10.00 mg/L to 100.0 mg/L, the *k* increased to 2.7 times, showing that nanoparticles were more prone to reunite.

#### 3.2.4. Sedimentation of Nano-Al_2_O_3_ in Natural Seawater

**Figure 7:**
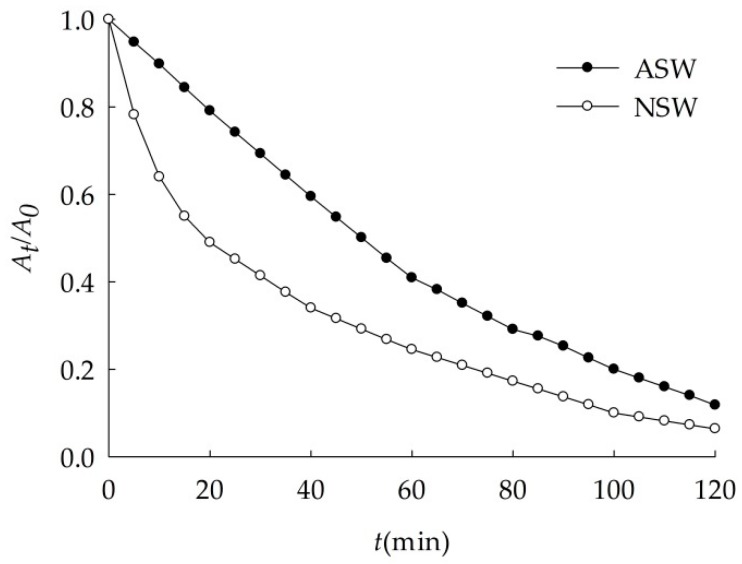
Sedimentation of nano-Al_2_O_3_ in NSW and ASW (*S* = 29.8, pH = 7.95).

Sedimentation of nano-Al_2_O_3_ in natural seawater was faster than that in artificial seawater, as shown in Figure 7 and Table 9, and *k* was 0.021 and 0.019 min^−1^ respectively. This is due to the difference of chemical composition between ASW and NSW. That is because nanoparticles could absorb and complex with nutrients, organic components, heavy metals, and other substances in seawater [37] and became bigger particles. The study showed that [38], the chelation of natural organic matter and the surface Cu^2+^ caused the reunion of nano-CuO in the water, while the reunion and settlement of nano-ZnO were mainly controlled by inorganic salt, and nano-CeO_2_ aggregated with iron and aluminum colloids to settle [39]. For lacking adjacent atoms around the surface, nano-Al_2_O_3_ were unsaturated and could be easily stabilized by combining with other atoms, so they showed high chemical activity. As shown in Figure 7, when nano-Al_2_O_3_ enters natural seawater in the first 1 h, other substances in seawater, especially colloids, would rapidly be adsorbed to its surface, making its effective radius rapidly increase. Therefore, *A_t_*/*A*_0_ value decreases faster than that in artificial seawater. However, some studies have put forward the opposite view that the dissolved organic matters in groundwater improves the dispersibility of nanoparticles [40]. The results showed that the factors affecting the sedimentation of different nanoparticles were varied.

## 4. Conclusions

It was an effective analysis method for nano-Al_2_O_3_ that ultrasonic dispersed the suspension for 30 min at 30 °C and 105 W power, before determining its absorbance at 340 nm. Method detection limit was 0.93 μg/L, standard addition recovery was 97.4–106.4%, the precision of was 2.2–5.5%.

In artificial seawater, the sedimentation rate of nano-Al_2_O_3_ associated with pH. When pH approached to the isoelectric point of nano-Al_2_O_3_ at 7.10, the particles were prone to aggregate. The greater pH deviated from the isoelectric point, the more stable the suspension. Furthermore, sedimentation rate of nano-Al_2_O_3_ was positively correlated with salinity and concentration of suspension, it is a bit higher in natural seawater than that in artificial seawater by 10.5%.

All the results revealed that nano-Al_2_O_3_ may be removed from the water in the process of the migration from river to sea.

## Figures and Tables

**Table 1:** Ultrasonic dispersion experiments of nano-Al_2_O_3_ ASW suspensions.

No.	Constant Conditions	Varied Conditions
1	P = 105 W, T = 30 °C	*t* (min) = 0, 10, 20, 30, 40, 50
2	*t* = 30 min, T = 30 °C	P (W) = 60, 75, 90, 105, 120
3	P = 105 W, *t* = 30 min	T (°C) = 20, 25, 30, 35, 40, 45, 50

**Table 2:** Experiments on the sedimentation of nano-Al_2_O_3_ ASW suspensions.

No.	Constant Conditions.	Varied Conditions
1	*S* = 31.5, C (Al_2_O_3_, mg/L) = 100.0	pH = 6.57, 7.31, 8.03, 8.97
2	pH = 8.00, C (Al_2_O_3_, mg/L) = 100.0	*S* = 0.2, 10.0, 20.0, 31.5
3	*S* = 31.5, pH = 8.00	C (Al_2_O_3_, mg/L) = 10.00, 30.00, 50.00, 80.00, 100.0

**Table 3:** Recovery of nano-Al_2_O_3_ by UV spectrophotometry.

Initial Concentration (mg/L)	Additive (mg/L)	Result (mg/L)	Recovery (%)
	5.00	14.90	106.4
9.00	10.00	19.82	104.3
	15.00	25.13	104.7
	10.00	28.44	97.4
19.21	20.00	39.93	101.8
	30.00	50.13	101.9
	20.00	59.90	99.0
40.52	30.00	70.91	100.6
	40.00	81.20	100.8

**Table 4:** Accuracy of UV spectrophotometry.

Actual Concentration (mg/L)	Testing Concentration (mg/L)						RSD (%)	Relative Deviation (%)
	1	2	3	4	5	6		
10.00	8.49	8.25	8.73	8.83	8.97	8.49	3.1	13.7
50.00	49.50	51.90	48.05	54.08	51.91	46.60	5.5	0.7
100.0	100.2	105.7	100.9	104.0	102.6	100.4	2.2	2.3

RSD: relative standard deviation.

**Table 5:** Comparison of gravimetry and UV spectrophotometry.

Actual Concentration (mg/L)	Gravimetry		UV Spectrophotometry	
	Testing Concentration (mg/L)	Relative Deviation (%)	Testing Concentration (mg/L)	Relative Deviation (%)
10.00	7.12	26.0	8.97	13.2
	6.57		8.25	
	8.51		8.83	
50.00	45.12	13.0	49.50	2.2
	41.06		51.91	
	44.32		51.90	
100.0	88.96	9.3	100.2	0.5
	90.68		100.9	
	92.34		100.4	

**Table 6:** Dynamics fitting of nano-Al_2_O_3_ sedimentation in ASW with different pH.

pH	*k* (min^−1^)	*r*
6.57	0.016	0.9970
7.31	0.021	0.9955
8.03	0.019	0.9954
8.97	0.010	0.9979

**Table 7:** Dynamics fitting of nano-Al_2_O_3_ sedimentation in ASW with different salinity.

*S*	*k* (min^−1^)	*r*
0.2	0.006	0.9970
10.0	0.010	0.9972
20.0	0.013	0.9970
31.5	0.019	0.9954

**Table 8:** Dynamics fitting of nano-Al_2_O_3_ sedimentation with different concentration in ASW.

C (Al_2_O_3_, mg/L)	*k* (min^−1^)	*r*
10.00	0.007	0.9943
30.00	0.008	0.9980
50.00	0.011	0.9961
80.00	0.015	0.9974
100.0	0.019	0.9954

**Table 9:** Dynamics fitting of nano-Al_2_O_3_ sedimentation in NSW and ASW.

Medium	*k* (min^−1^)	*r*
NSW	0.021	0.9957
ASW	0.019	0.9954

## References

[1] Maynard A.D., Aitken R.J., Butz T., Colvin V., Donaldson K., Oberdorster G., Philbert M.A., Ryan J., Seaton A., Stone V. (2006). Safe handling of nanotechnology. Nature.

[2] Wiesner M.R., Lowry G.V., Alvarez P., Dionysiou D., Biswas P. (2006). Assessing the risks of manufactured nanomaterials. Environ. Sci. Technol..

[3] Murali M., Suganthi P., Athif P., Bukhari A.S., Mohamed H.E.S., Basu H., Singhal R.K. (2017). Histological alterations in the hepatic tissues of Al_2_O_3_ nanoparticles exposed freshwater fish Oreochromis mossambicus. J. Trace Elem. Med. Biol..

[4] Li J., Hu X., Chen Q., Yin D. (2011). Ecotoxicology of nanomaterials on aquatic organisms. Environ. Chem..

[5] Matranga V., Corsi I. (2012). Toxic effects of engineered nanoparticles in the marine environment: Model organisms and molecular approaches. Mar. Environ. Res..

[6] Canesi L., Corsi I. (2016). Effects of nanomaterials on marine invertebrates. Sci. Total Environ..

[7] Baker T.J., Tyler C.R., Galloway T.S. (2014). Impacts of metal and metal oxide nanoparticles on marine organisms. Environ. Pollut..

[8] Brayner R., Dahaumane S.A., Yepremian C., Djediat C., Meyer M., Coute A., Fievet F. (2010). ZnO nanoparticles: Synthesis, characterization, and ecotoxicological studies. Langmuir.

[9] Pakrashi S., Kumar D., Iswarya V., Bhuvaneshwari M., Chandrasekaran N., Mukherjee A. (2014). A comparative ecotoxicity ananlysis of α- and γ- phase aluminium oxide nanoparticles towards a freshwater bacterial isolate Bacillus licheniformis. Bioprocess Biosyst. Eng..

[10] Wang D. (2012). Development and use of nano-alumina. Adv. Ceram..

[11] Doskocz N., Affek K., Zaleska-Radziwill M. Effects of aluminium oxide nanoparticles on bacterial growth. Proceedings of the E3S Web of Conferences, 9th Conference on Interdisciplinary Problems in Environmental Protection and Engineering (EKO-DOK).

[12] Wiesner M.R., Lowry G.V., Jones K.L., Hochella M.F., Di Giulio R.T., Casman E., Bernhardt E.S. (2009). Decreasing uncertainties in assessing environmental exposure, risk, and ecological implications of nanomaterials. Environ. Sci. Technol..

[13] Lin P., Guo L., Chen M. (2014). Adsorption and fractionation of thorium and protactinium on nanoparticles in seawater. Mar. Chem..

[14] Van Hoecke K., De Schamphelaere K.A.C., Van der Meeren P., Smagghe G., Janssen C.R. (2011). Aggregation and ecotoxicity of CeO_2_ nanoparticles in synthetic and natural waters with variable pH, organic matter concentration and ionic strength. Environ. Pollut..

[15] Omar F.M., Aziz H.A., Stoll S. (2014). Aggregation and disaggregation of ZnO nanoparticles: Influence of Ph and adsorption of Suwannee River humic acid. Sci. Total Environ..

[16] Guo W., Wang W., Guo X., Lv X. (2009). Research on the stability of nanoTiO_2_ in the system of water dispersion. Appl. Chem. Ind..

[17] Zhou X., Li W., He L. (2005). Dispersion stability of nanoparticles and their assessment methods. Mater. Prot..

[18] Aureli F., D’Amato M., De Berardis B., Raggi A., Turco A.C., Cubadda F. (2012). Investigating agglomeration and dissolution of silica nanoparticles in aqueous suspensions by dynamic reaction cell inductively coupled plasma-mass spectrometry in time resolved mode. J. Anal. At. Spectrom..

[19] Chinnapongse S.L., MacCuspie R.I., Hackley V.A. (2011). Persistence of singly dispersed silver nanoparticles in natural freshwaters, synthetic seawater, and simulated estuarine waters. Sci. Total Environ..

[20] Sikder M., Lead J.R., Chandler G.T., Baalousha M. (2018). A rapid approach for measuring silver nanoparticle concentration and dissolution in seawater by UV-Vis. Sci. Total Environ..

[21] Yegin B.A., Lamprecht A. (2006). Lipid nanocapsule size analysis by hydrodynamic chromatography and photon correlation spectroscopy. Int. J. Pharm..

[22] Bidwell J.P., Spotte S. (1985). Artificial Seawaters: Formulas and Method.

[23] Li H. (2010). Comparison of several calculation methods of detection Limit. Chin. J. Spectrosc. Lab..

[24] GB 17378.4-2007 (2007). The Specification for Marine Monitoring: Part 4: Seawater Analysis.

[25] Abel J.S., Stangle G.C., Schilling C.H., Aksay I.A. (1994). Sedimentation in flocculating colloidal suspensions. J. Mater. Res..

[26] Singh B.P., Menchavez R., Takai C., Fuji M., Takahashi M. (2005). Stability of dispersions of colloidal alumina particles in aqueous suspensions. J. Colloid Interface Sci..

[27] Chen J., Li N., Fang J. (2012). Dispersion and deposition of the suspensions of TiO_2_ nanoparticles in the presence of surfactant. J. Zhejiang Univ. Technol..

[28] Chen Q., Wang J., Li W., Yin Z. (2008). Influence of dispersants on dispersion stability of super-fine alumina particles suspension. China Powder Sci. Technol..

[29] Wu Q., Yang C., Hu X., Dang Z., LI Y. (2012). Influences of environmental factors on aggregation of titanium dioxide nanoparticles. Acta Sci. Circumst..

[30] Mudunkotuwa I.A., Grassian V.H. (2010). Citric acid adsorption on TiO_2_ nanoparticles in aqueous suspensions at acidic and circumneutral pH: Surface coverage, surface speciation, and its impact on nanoparticle-nanoparticle interactions. J. Am. Chem. Soc..

[31] Jia Y., Kanno Y., Xie Z.P. (2003). Fabrication of alumina green body through gelcasting process using alginate. Mater. Lett..

[32] Akhondi H., Taheri-Nassaj E., Sarpoolaky H., Taavoni-Gilan A. (2009). Gelcasting of alumina nanopowders based on gelation of sodium alginate. Ceram. Int..

[33] Domingos R.F., Tufenkji N., Wilkinson K.J. (2009). Aggregation of titanium dioxide nanoparticles: Role of a fulvic acid. Environ. Sci. Technol..

[34] Zhang Y., Chen Y.S., Westerhoff P., Hristovski K., Crittenden J.C. (2008). Stability of commercial metal oxide nanoparticles in water. Water Res..

[35] French R.A., Jacobson A.R., Kim B., Isley S.L., Penn R.L., Baveye P.C. (2009). Influence of ionic strength, pH, and cation valence on aggregation kinetics of titanium dioxide nanoparticles. Environ. Sci. Technol..

[36] Dai M., Martin J.M., Cauwet G. (1995). The significant role of colloids in the transport and transformation of organic carbon and associated trace metals (Cd, Cu and Ni) in the Rhône delta (France). Mar. Chem..

[37] Hua J., Yuan J., Sheng G. (2016). Aggregation and sedimentation of metal oxides nanoparticles in aquatic environment. Environ. Sci. Technol..

[38] Van Koetsem F., Verstraete S., Vander Meeren P., Du Laing G. (2015). Stability of engineered nanomaterials in complex aqueous matrices: Settling behavior of CeO_2_ nanoparticles in natural surface waters. Environ. Res..

[39] Johnson R.L., Johnson G.O., Nurmi J.T., Tratnyek P.G. (2009). Natural organic matter enhanced mobility of nano zerovalent iron. Environ. Sci. Technol..

[40] Pelley A.J., Tufenkji N. (2008). Effect of particle size and natural organic matter on the migration of nano-and microscale latex particles in saturated porous media. J. Colloid Interface Sci..

